# FASCAPLYSIN as a Specific Inhibitor for CDK4: Insights from Molecular Modelling

**DOI:** 10.1371/journal.pone.0042612

**Published:** 2012-08-14

**Authors:** Muhammad Imtiaz Shafiq, Thomas Steinbrecher, Ralf Schmid

**Affiliations:** 1 Department of Biochemistry, University of Leicester, Leicester, United Kingdom; 2 Institute of Chemistry, University of the Punjab, Lahore, Pakistan; 3 Institut für Physikalische Chemie, Abteilung für Theoretische Chemische Biologie, Karlsruher Institut für Technologie, Universität Karlsruhe, Karlsruhe, Germany; Universität Erlangen-Nürnberg, Germany

## Abstract

Cyclin-dependent kinases (CDKs) play a key role in the cell cycle and are important anti-cancer drug targets. The natural product fascaplysin inhibits CDK4 with surprising selectivity (IC_50_ = 0.4 µM) compared to the close homolog CDK2 (IC_50_ = 500 µM). Free energy calculations of the positively charged fascaplysin and an uncharged iso-electronic derivative in the CDK2 and CDK4 inhibitor complexes indicate that the positive charge of fascaplysin is crucial for selectivity. This finding will guide further improvements in the design of fascaplysin-based selective inhibitors for CDK4.

## Introduction

Cyclin dependent kinases (CDKs) are a group of protein kinases which regulate different stages of the eukaryotic cell cycle [1]–[4]. CDKs are also involved in the control of gene transcription, the processes that integrate extracellular and intracellular signals for the coordination of the cell cycle in response to environmental change, and apoptosis [2], [5], [6]. Activation of CDKs usually occurs via phosphorylation of specific threonine residues by the CDK-activating kinase and binding to a cyclin protein. CDK4 plays a central role in the regulation of the G_0_–G_1_ phase of the cell and is required for the G_1_/S phase transition. CDK4 inactivates the retinoblastoma protein (pRb) by phosphorylation. pRb is a negative regulator of the E2F family of transcription factors [7], hence phosphorylation of pRb results in the release of transcription factors which activate the expression of the S-phase genes. This process enables the cell to pass through the restriction point and results in the onset of the S-phase [7]–[9]. Cell cycle regulators are frequently mutated in human cancers and due to their central role in G_1_ regulation CDKs offer attractive targets for therapeutic inhibition [10]–[12]. The work of Yu *et al.* [13] and Landis *et al.* [14] suggests that inhibition of CDK4 might benefit patients with ErbB-2 initiated breast cancers [12]. The CDK4/CyclinD1 complex as an anti-cancer drug target has been further validated in MCF-7 breast cancer cells [15].

**Figure 1:**
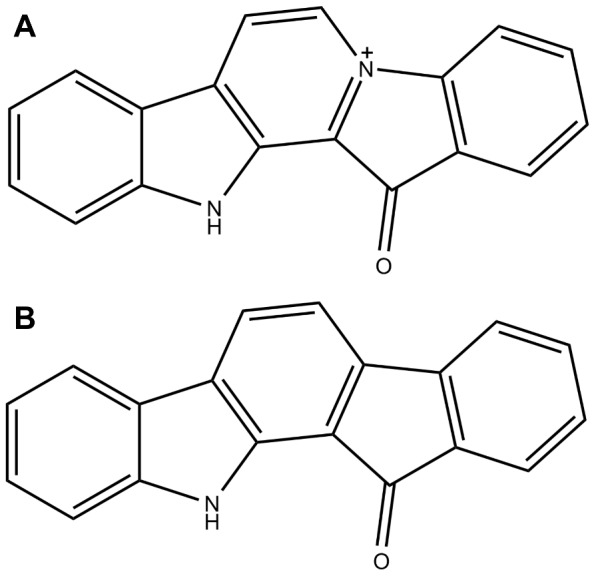
Molecular structures of fascaplysin (FAS) and “carbofascaplysin” (CRB). Fascaplysin (**A**) is a natural product from the sponge *Fascaplysinopsis Bergquist*. Isoelectronic substitution of the positively charged nitrogen with a carbon atom leads to an electrically neutral derivative (**B**), which for the ease of discussion we refer to as “carbofascaplysin”.

**Figure 2:**
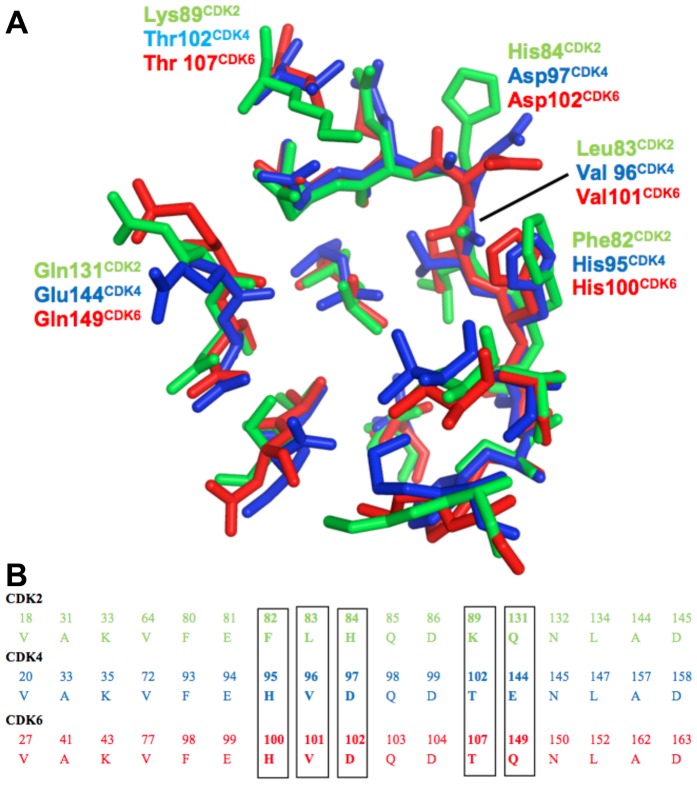
Active site conservation in CDK2, CDK4 and CDK6. (**A**) Structural overlay of active site residues for CDK2 (green, PDB-ID: 1FIN), CDK4 (blue, PDB-ID: 2W96) and CDK6 (red, PDB-ID: 1G3N). (**B**) Corresponding sequence alignment of the active site residues, the colour scheme is as in (**A**).

More than 20 small molecule inhibitors for CDKs are in clinical trials (for recent reviews see [16]–[19]). For example, Flavopiridol (Alvocidib) is in clinical development for the treatment of different metastatic cancers [20]–[22]. R-Roscovitine (Seliciclib, CYC202) inhibits CDK2, CDK7 and CDK9 [23] and is also in clinical trials. To avoid side effects, high selectivity is desirable, though difficult to achieve as the ATP binding site of the human kinome is well conserved [24], [25]. Recently, selective inhibitors for CDK4 have gained substantial interest [26], [27]. For example the orally active small molecule PD0332991, which induces G_1_ arrest in primary myeloma cells, prevents tumor growth by specific inhibition of CDK4/6 and is now in Phase 2 clinical trials [28], [29]. The natural compound fascaplysin (Figure 1), originally isolated from the sponge *Fascaplysinopsis Bergquist* [30], is a kinase inhibitor with enticing selectivity for CDK4 (IC_50_∼0.4 µM) relative to the close homolog CDK2 (45% sequence identity, IC_50_∼500 µM), and also shows approximately eightfold selectivity over CDK6 (68% sequence identity, IC_50_∼3.4 µM) [31]. Approximating the dissociation constant K_D_ with IC_50_ and using the relation ΔG^0^ = −RTlnK_D_, the difference in the free energy of binding between the CDK4/fascaplysin and CDK2/fascaplysin complexes can be calculated to 4.2 kcal/mol. Considering the close structural similarity of the active sites of CDK2, CDK4 and CDK6, and the relatively small size (Mw = 306.75) and rigid structure of fascaplysin, the observed selectivity is remarkable. Chemically, fascaplysin is a planar, aromatic compound with no freely rotatable single bonds. It comprises five condensed rings, the central ring includes a positively charged imminium nitrogen. An indol-NH and a carbonyl can act as H-bond donor and H-bond acceptor, respectively. The H-bond donor and H-bond acceptor in fascaplysin are oriented in parallel spaced at ∼2.6 Å, a feature shared with other kinase inhibitors. The fascaplysin framework has been used to synthesise a series of selective CDK4 inhibitors [31]–[37], though in most cases selectivity was partially lost in the re-design process. So what are the features that could explain the remarkable selectivity of fascaplysin? There is a considerable amount of structural information on CDKs available to help addressing this question. More than 100 CDK2 structures in complex with small molecules are deposited in the protein databank. However, compared to CDK2, structural information on CDK6 and CDK4 with inhibitors bound is scarce, in fact the first CDK4 structures have only been published recently [38], [39]. Most residues in the active sites of CDK2, CDK4 and CDK6 are remarkably conserved (Figure 2). A key difference is the presence of a histidine residue in CDK4/6 (His95^CDK4^ and His100^CDK6^) while CDK2 comprises a phenylalanine (Phe82) in the equivalent position. The His95^CDK4^/His100^CDK6^ side-chain is in a position where it potentially can donate or accept a H-bond from an inhibitor. Other differences are in Val96^CDK4^ and Val101^CDK6^ corresponding to Leu83^CDK2^. This residue is capable of forming H-bonds to inhibitors with both backbone NH and carbonyl group, but as its side chain is pointing away from the binding site and is not in direct contact with inhibitors the Val/Leu variation appears to be less relevant for selectivity. Other differences in the binding site are residues Thr120^CDK4^ and Thr107^CDK6^, these threonines correspond to Lys89^CDK2^. The negatively charged residues Asp97^CDK4^ and Asp102^CDK6^ have His84^CDK2^ in the equivalent position, and finally glutamate Glu144^CDK4^ is corresponding to Gln131^CDK2^ and Gln149^CDK6^ – the latter being the only position where CDK4 and CDK6 have different residues. Interestingly, in all three of these positions CDK4 gains a negative charge relative to CDK2. The potential role of charge as a determinant of CDK4 inhibitor specificity has been pointed out originally by McInnes et al. [40] and more recently by Mascarenhas et al. [41]. In this work, we have studied this example of charge-determined protein-ligand interactions using a variety of methods from the molecular modelling and drug design fields.

The binding of inhibitors to protein receptors with high affinity and specificity is central to structure-based drug design applications. The quest for the calculation of binding affinities remains one of the main goals of modern computational biophysical methods [42]–[48]. The most accurate methods for calculating binding free energies are based on molecular dynamics simulations which predict the physical properties of the protein-ligand complexes based on atomistic structural models. The energetic consequences of small structural changes in inhibitor complexes have been successfully studied using thermodynamic integration [49]–[54]. An added benefit of TI calculations, as compared to empirical ligand docking algorithms is that the former include accurate estimates of binding entropy as well as enthalpy, based on rigorous statistical thermodynamics. In this work, we specifically address the contribution of the positive charge of fascaplysin (FAS, see Figure 1A) to selectivity by applying thermodynamic integration calculations. *In silico*, fascaplysin can be modified easily by the iso-electronic substitution of the positively charged nitrogen to a charge neutral carbon atom, resulting in a compound, which for clarity and simplicity we refer to as carbofascaplysin (CRB, indolo-[1,2-*c*]-fluorene-9-one, see Figure 1B). By calculating the energetic effect of this substitution for the protein-inhibitor complexes of both CDK2 and CDK4, we can quantify the impact of the positive charge of fascaplysin on its specificities towards CDK2 and CDK4.

## Methods

### Structure preparation, homology modelling and ligand docking

The X-ray crystal structure of CDK2 in its active form (PDB-ID: 1FIN [55]) was used as starting structure for molecular modelling. For CDK4 ligand docking, molecular dynamics simulations and free energy calculations a different strategy was employed. As the five published X-ray structures for CDK4 [38], [39] have sections missing and are in an inactive state, a ‘hybrid’ model was constructed in modeller v9.1 [56]. In this ‘hybrid’ model the core of the structure is based on the experimentally solved CDK4 structure 2W96, but information from CDK2 (PDB-ID: 1FIN) is used for missing regions and the positioning of the activation loop. ProSa-web [57] and WhatCheck [58] were used for model validation. The coordinates of the ‘hybrid model’ are provided as supporting information (File S1). The inhibitor fascaplysin (FAS) and the hypothetical compound ‘carbofascaplysin’ (CRB) were built in Hyperchem 8.0 [59], and energy minimised prior to docking. Ligand docking calculations were performed in GOLD v4.1 [60] using the ChemScore scoring function with the binding site defined with a radius of 12 Å around the backbone-N of Leu83 for CDK2 and Val96 for CDK4. PyMOL and VMD were used for molecular visualisation. [61], [62]

### Molecular dynamics simulations

All molecular dynamics simulations were performed using the Amber 10 package [63] with the ff99SB [64] force field for proteins and GAFF [65] for ligands. RESP partial charges for the two ligands FAS and CRB were derived using GAUSSIAN03 [66] at the HF/6-31G* theory level and the antechamber program. CDK2 and CDK4 were solvated in a cubic solvent box so that the distance between every solute atom and the box boundary was at least 12 Å and neutralised by adding counter ions. Water molecules were treated using the TIP4P-Ew water model, a re-parameterization of TIP4P [67] with Ewald summation [68]; five buried crystal waters for CDK2 (PDB-ID 1FIN) and four crystal waters present in equivalent positions in CDK4 were kept in the simulations. Before the simulations the systems were energy minimized, initially by steepest descent followed by conjugate gradient minimization. Then the energy-minimised complexes were equilibrated by 50 ps heating from 0 K to 300 K followed by 50 ps density equilibration, both with weak restraints for all protein residues (2.0 kcal/mol Å^−2^) and 500 ps constant pressure equilibration. Production runs were run for 5 ns with 2 fs time steps. For all simulations the SHAKE algorithm was used to constrain bonds between hydrogens and heavy atoms [69], Langevin dynamics were used for temperature control. Amber tools were used for the analysis of the MD runs, for example the presence or absence of H-bonds was tested using the ptraj hbond command with default settings (heavy atom distance < = 3.0 Å, donor-H-acceptor angle > = 135°)

#### Thermodynamic Integration

**Figure 3:**
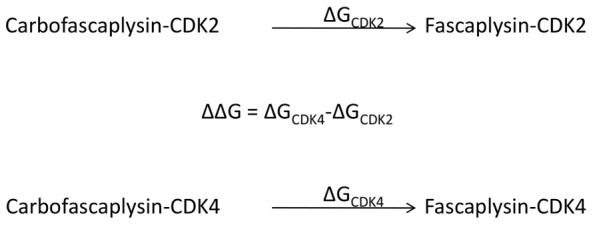
Thermodynamic scheme for calculating the contribution of inhibitor charge to the free energy difference in CDK4/fascaplysin and CDK2/fascaplysin complexes. The energetic contribution of inhibitor charge to specificity is calculated as difference from two independent steps, the transformation of the CDK4/carbofascaplysin complex to CDK4/fascaplysin and the transformation of the CDK2/carbofascaplysin complex to CDK2/fascaplysin.

Thermodynamic integration (TI) estimates the free energy changes between two states A and B by coupling them via an additional, non spatial coordinate lambda (λ) [70]. TI simulations were carried out for transformation of CRB to FAS in CDK2, CDK4 and water. Linear mixing of the potential functions *V*_0_ and *V*_1_ was used, where *V*_0_ and *V*_1_ correspond to the potential function for the CRB (λ = 0) and FAS (λ = 1) states, respectively. Hence, the combined potential function *V*(λ) is a function of the perturbation variable λ and can be described as *V*(λ) = (1−λ) *V*_0_+λ *V*_1_. Since mainly electrostatic changes were studied, it was not necessary to use soft core potentials for simulation stability, as e.g. in [71]. Note that with this TI setup, the total system charge changes during a single transformation step, while overall charge neutrality for the thermodynamic cycle is of course maintained. Charge-change TI calculations involve some additional practical challenges when compared to charge neutral ones, as electrostatic interactions are strong and long-ranged, leading to potential convergence problems. Nevertheless, the PME long-range electrostatics treatment used here allows for simulations of such net-charge changes [72]. The alternative of simultaneously generating/removing a counter ion for an overall charge-neutral transformation poses equally large sampling problems and was avoided here, as is commonly done in similar studies [73], [74]. The thermodynamic integration simulations were run for 19 λ-points/windows (λ = 0.05 to λ = 0.95, 5 ns each window). Each 5 ns simulation was divided into 25 steps of 200 ps. For each step the d*V*/dλ integral was solved numerically by computing the weighted average of 19 evenly spaced δ*V*/δλ values (0.05, 0.10, … 0.95). λ-points were weighted by 0.05 each, with the exception of λ = 0.05 and λ = 0.95, which were weighted at 0.075 to extrapolate to the end points. Linear extrapolation and the trapezoid rule were used for integration. ΔΔG^0^ for the relative stabilisation of CRB/FAS in CDK4/CDK2 were calculated as illustrated in Fig. 3.

## Results and Discussion

### Homology modelling and ligand docking

CDK4 had escaped structural characterisation by X-ray crystallography for a long time, but in 2009 Day et al. and Takaki et al. achieved a major breakthrough and solved its structure in complex with cyclin D1 [38] and cyclin D3 [39], respectively. These experimentally determined CDK4 structures are proposed to represent an intermediate, not fully activated state [38], [39] and none of the as yet published structures contains a small molecule inhibitor in the ATP binding site. Before experimentally determined CDK4 structures became available, CDK4 homology models based on experimentally determined structures of CDK 2 and/or CDK6 were commonly used for computational studies such as ligand docking e.g. [34], [75]–[77] and molecular dynamics simulations [78]–[80]. Most small molecule CDK4 inhibitors are competitive inhibitors for ATP [81] and target the active form of CDK4. Hence, CDK4 homology models representing the active form still have been used in recent ligand docking studies, despite the availability of experimentally determined CDK4 structures. [41], [82] To take advantage of the new X-ray structures we opted for a ‘hybrid model’ strategy for studying the binding behaviour and selectivity of fascaplysin. The core of the ‘hybrid model’ for CDK4 was built using the CDK4 structure 2W96 as template, but the modelling strategy also made use of an active form CDK2 structure (PDB-ID: 1FIN) for modelling the T-loop and to impose an active conformation on the C-helix of CDK4. ProSa-Web Z-scores for the ‘hybrid model’ and the CDK4 and CDK2 templates are −7.84, −7.96 and −7.12, respectively, indicating that the modelling strategy has not introduced any significant packing problems. The rmsd between the active form ‘hybrid model’ and the experimentally determined CDK4 structure (PDB-ID: 2W9F) is 1.5 Å, this is close to the 1.2 Å found for comparing the active (PDB-ID: 1FIN) and inactive form (PDB-ID: 2R3I) of CDK2. FAS and CRB were docked into both, CDK2 and CDK4, using the GOLD package. GOLD treats ligands as fully flexible and allows the user to assign flexibility to a limited set of receptor residues. The best-scoring docking poses for all four systems investigated show key features known from experimentally determined CDK2/inhibitor X-ray structures such as hydrogen bonding in the hinge region. FAS and CRB docking poses are characterised by two H-bonds involving the backbone NH and carbonyl of the hinge residues Leu83^CDK2^ and Val96^CDK4^. No significant difference was found in the docking scores of both compounds with both CDK2 and CDK4 (ChemScores are in the range of 29 to 31 for CDK2, and 31 to 34 for CDK4, respectively). So while the ligand docking study generates typical kinase inhibitor binding poses, it can not explain why fascaplysin preferably binds to CDK4 rather than CDK2.

**Figure 4:**
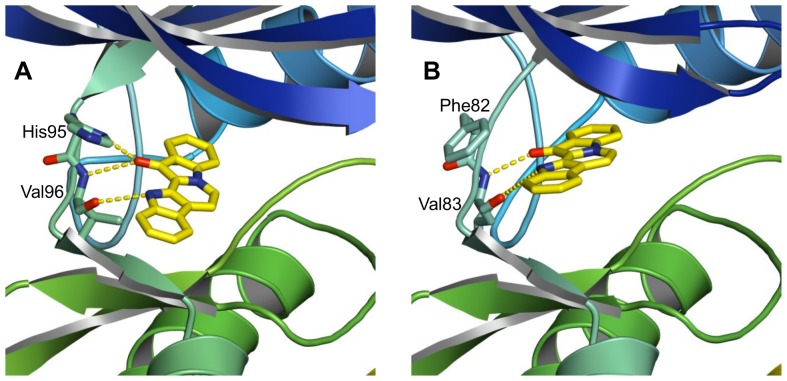
Predicted binding modes for the fascaplysin/CDK4 and the fascaplysin/CDK2 complexes. The predicted binding modes for CDK4 (**A**) and CDK2 (**B**) show two hydrogen bonds between NH and carbonyl groups of fascaplysin and the backbone carbonyl and NH of Val96 in CDK4 and of Leu83 in CDK2, respectively. In the CDK4/fascaplysin binding mode an additional hydrogen bond between the Nδ-H of the His95^CDK4^ imidazole side chain and fascaplysin is possible.

A key difference between the CDK2 and CDK4 poses involves the equivalent residues His95^CDK4^ and Phe82^CDK2^. In principle three different ‘species’ of His95^CDK4^ have to be considered: His95^CDK4^ could have a positively charged imidazole side chain and there are two uncharged species with either Nδ or Nε of the imidazole ring bearing a hydrogen. We did not consider a positively charged imidazole side chain as this would unfavourably interact with the positively charged fascaplysin. However, alternative positioning of hydrogens in His95^CDK4^ (Nδ-H and Nε-H) was considered in the ligand docking process. ChemScores were by a small margin higher for the His95^CDK4^Nδ-H/fascaplysin complex (33.4 compared to 31.3 for His95^CDK4^Nε-H/fascaplysin complex) indicating a slight preference for the side-chain conformation in which the Nδ-hydrogen of the imidazol ring forms an additional H-bond to the carbonyl of FAS and CRB, respectively. This conformation is different from the His95 conformation found in the experimentally determined CDK4 structures, but such a conformational change could occur upon ligand binding, when the alternative His95^CDK4^ side chain conformation is stabilised by the interaction with the inhibitor (Figure 4A). The idea of His95 as a key player for CDK4 specificity is supported by the notion that CDK6 also has a histidine residue in the equivalent position. Any energetic contribution of the additional His95-Nδ H-bond to the free energy of binding should also feature in CDK6, and indeed the IC_50_ of CDK6/fascaplysin is, while being ∼8 times higher than CDK4/fascaplysin, still ∼100 times lower than CDK2/fascaplysin. However, there is a problem with this notion, as if correct, the interaction in question should occur for most inhibitors, essentially for any ligand that forms a H-bond with the backbone NH of Val96^CDK4^. If His95^CDK4^ was indeed the key to the observed fascaplysin CDK4 specificity we would expect this to be rather generic feature, rendering most CDK inhibitors more specific for CDK4 as CDK2. This is however not the case and hence it is unlikely that the difference between His95^CDK4^ and Phe82^CDK2^ can account fully for the differential binding of fascaplysin.

The inaccuracy of docking scoring functions for estimating free energies of binding is a major short coming of typical ligand docking approaches [83]–[85]. To obtain more accurately calculated values for free energies of binding thermodynamic integration was used. A key feature of fascaplysin is its positive charge. Docking scoring functions are limited in accounting for long-range electrostatic interactions; Thermodynamic Integration however describes long-range electrostatic interactions more accurately as the Particle Mesh Ewald method for calculating electrostatic energy terms also incorporates orientation polarisation effects (conformational response to charge). The Thermodynamic Integration approach was used to specifically address the role of charge as a determinant of CDK4 inhibitor selectivity comparing the charge stabilisation in CDK2/CRB->CDK2/FAS and His95 Nε-H CDK4/CRB->CDK4/FAS complexes [40], [41]. To better account for protein flexibility in response to inhibitor binding a series of six 5 ns molecular dynamics simulation was performed. The comparison between runs with all four inhibitor-protein complexes, FAS and CRB as inhibitors, and CDK2 and CDK4 (both His95 conformers) as receptors, allows the investigation of conformational change in response to changes of charge of inhibitors.

### Molecular dynamics simulations

**Figure 5:**
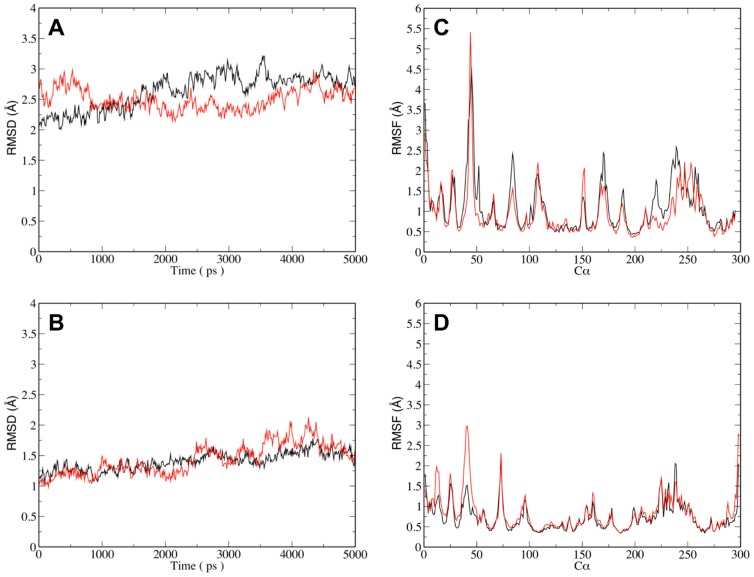
5 ns MD simulations for CDK4 and CDK2 complexed with fascaplysin and carbofascaplysin. (**A**) Comparison of backbone rmsd (relative to the energy minimised starting structure) as a function of time for the CDK4/fascaplysin (red) and CDK4/carbofascaplysin (black) simulations (**B**) Comparison of rmsd (relative to the energy minimised starting structure) for the CDK2/fascaplysin (red) and CDK2/carbofascaplysin (black) simulations. (**C**) Cα RMSF values for the simulations shown in (**A**). The peak for residues 42–48 corresponds to a flexible poly-glycine region not present in CDK2 (**D**) Cα RMSF values for the in the simulations shown in (**C**).

Before endeavouring on TI runs the systems corresponding to the λ = 0 and λ = 1 endpoints, *i.e.*, CDK2/fascaplysin and CDK2/carbofascaplysin, CDK4-His95^CDK4^-Nε-H/carbofascaplysin and CDK4-His95^CDK4^-Nε-H/fascaplysin, and for comparison CDK4-His95^CDK4^-Nδ-H/fascaplysin and CDK4-His95^CDK4^-Nδ-H/carbofascaplysin were tested for stability (Figure 5). The average rmsd (compared to the energy minimised starting structure) over 5 ns CDK2 simulations is less than 2 Å for both runs, the average rmsd for the respective CDK4 simulations is slightly higher. This higher value is not unexpected as the CDK4 structure used for simulations is the ‘hybrid model’ as described in the materials and methods section while a experimentally determined high resolution X-ray structure was used for CDK2. It is however lower or similar to rmsds that have been reported in MD simulations using CDK4 homology models previously [41], [78]–[80]. Also, in comparison to CDK2 the CDK4 structure contains a flexible poly-Glycin loop comprising seven glycines (residues 42–48) not present in CDK2. These residues display relatively high Cα-RMSF values (Figure 5C) and contribute to the higher average rmsd.

Buried waters are often a concern in molecular dynamics simulations. If they are not transferred from an experimental structure they are often missed when generating the water box. Nine water molecules from the CDK2 X-ray structure (PDB ID: 1FIN) were kept for the MD simulations based on their “conservation” across a set of 21 CDK2 inhibitor structures with a resolution of 1.8 Å or better. Inherently, such an approach is more difficult for the CDK4 hybrid model, but based on the CDK4 structures solved by Day et al. [38] four buried water molecules were included in the CDK4 simulations. Compared to preliminary simulations which were not using waters from the experimental structures, the inclusion of these waters enhanced the stability of the simulations for both, CDK2 and CDK4 simulations (data not shown).

**Figure 6:**
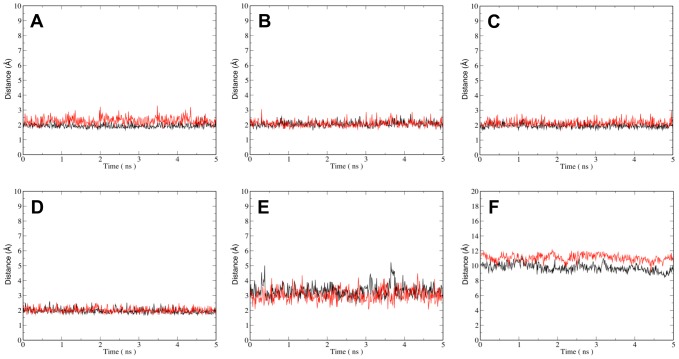
Binding of fascaplysin and carbofascaplyin in CDK4 and CDK2. (**A**) Distances between the carbonyl oxygen (black), and amide hydrogen (red) of V96^CDK4^ and the indoyl hydrogen and carbonyl oxygen of FAS, respectively. (**B**) Distances between the carbonyl oxygen (black) and amide hydrogen (red) of V96^CDK4^ and the indoyl hydrogen and carbonyl oxygen of CRB, respectively. (**C**) Distances between the carbonyl oxygen (black) and amide hydrogen (red) of L83^CDK2^ and the indoyl hydrogen and carbonyl oxygen of FAS, respectively. (**D**) Distances between the carbonyl oxygen (black) and amide hydrogen (red) of L83^CDK2^ and the indoyl hydrogen and carbonyl oxygen of CRB, respectively. (**E**) Distances between the His95^CDK4^ Nδ-H and the carbonyl oxygen of FAS (red) and CRB (black), respectively. (**F**) Distances between Lys89^CDK2^ amine-N and the N+/C of FAS (red) and CRB (black), respectively.

The ligand docking poses for fascaplysin and carbofascaplysin in CDK2 and CDK4 suggest that all four binding modes are rather similar with both ligands forming two hydrogen bonds to backbone carbonyl and NH of Val96^CDK4^ and Leu83^CDK2^, respectively. Molecular dynamics simulations allow studying these binding poses over time to give a dynamic picture. (Figure 6). The two H-bonds to the backbone are present in 97%, 100% and 100% of the simulation snapshots in CDK2/FAS, CDK4-His95^CDK4^-Nδ-H/FAS, and CDK4-His95^CDK4^-Nε-H/FAS, respectively. The six simulations also allow addressing the question of the involvement of specific residues in the selectivity of fascaplysin to CDK4 by comparing residues which are different in CDK2 and CDK4 in the four simulations. The substitution of Phe82^CDK2^ with His95^CDK4^ in the equivalent position of CDK4 is one of the key differences in the active site. Ligand docking suggests that the side chain of His95^CDK4^ can form an additional polar interaction between with FAS and CRB, while Phe82^CDK2^ cannot play such a role. Monitoring the H-bonding between in CDK4/FAS and CDK4/CRB, reveals that indeed this takes place, but only in approximately 46% (CDK4/FAS) and 28% (CDK4/CRB) of the simulation time (Figure 6E).

Other residues of interest for CDK4 selectivity, originally proposed by McInnes et al, are Thr102^CDK4^ and Glu144^CDK4^ where the corresponding CDK2 residues are Lys89^CDK2^ and Gln131^CDK2^ in CDK2 [40]. For both residues the formal charge in CDK2 is increased by one. The positive charge of Lys89 in CDK2 could have a destabilising effect on binding of FAS in CDK2, and hence contribute to the different binding properties of CDK2 and CDK4. Interestingly, the average distances between Lys89-NZ and FAS-N2, and Lys89-NZ and CRB-C0 are 11.0 Å and 9.7 Å, respectively. The approximately 1.3 Å shorter distance in the CDK2/CRB complex indicates a conformational response of Lys89^CDK2^ to the positive fascaplysin charge (Figure 6F). Similarly, for the negatively charged residues Glu144^CDK4^ and Asp97^CDK4^ (corresponding to His84^CDK2^) conformational change to minimize the distance between FAS and the negatively charged side chains could occur. Monitoring the distances between OE1 and OE2 of Glu144^CDK4^, OD1 and OD2 of Asp97^CDK4^, and N2 and C0 of FAS and CRB, in all four CDK4 simulations does not support this idea. While the six molecular dynamics simulations allow monitoring structural differences potentially associated with differential binding, they do not provide quantitative information for this phenomenon.

### Thermodynamic Integration

**Figure 7:**
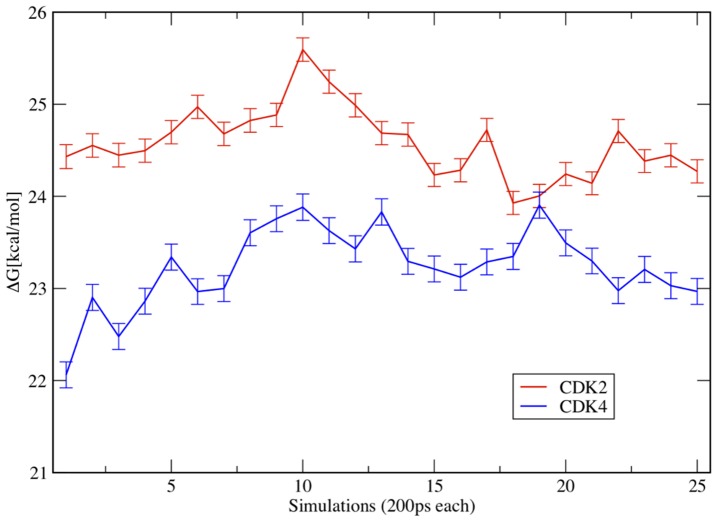
Thermodynamic Integration. Each point (200 ps window) represents the free energy “cost” of the carbofascaplysin to fascaplysin transformation in the CDK4 and CDK2 complex calculated from 19 values for λ. The difference (ΔΔG^0^) between the two plots quantifies the energetic contribution for selectivity that can be attributed to the positive fascaplysin charge.

Despite substantial progress in ligand docking one of the major limitations remains the inaccuracy of the scoring functions used for estimating binding energies. For a quantitative treatment of binding energies, computationally more accurate (and therefore computationally more expensive) methods are required. A method particularly well suited to calculate differences rather than absolute values of free energies of binding is thermodynamic integration. TI is best used in situations where small changes in structure correlate with relatively substantial changes in the free energy of binding. The preferential binding of fascaplysin to CDK4 with roughly 4.2 kcal/mol difference in the free energies of binding between the CDK4/fascaplysin and CDK2/fascaplysin complexes studied in this work clearly falls into this category. The role of positive charge on inhibitors for CDK4 specificity relative to CDK2 has been emphasized by McInnes et al. based on a two-unit increase in the formal charge of the binding pocket of CDK2 relative to CDK4 [40]. Such electrostatic interactions are long ranged and sensitive to large scale conformational motions, therefore extensive MD simulations need to be conducted to accurately capture their effect. To avoid these difficulties, TI studies are often limited to charge neutral transformations [71], [86]. In order to specifically quantify the effect of the positive charge of fascaplysin on differential binding to CDK2 and CDK4, the ‘energetic cost’ of mutating a neutral carbon atom (λ = 0) into a positively charged nitrogen (λ = 1) was calculated in the inhibitor complexes with CDK2 (ΔG^0^_CDK2_) and CDK4 (ΔG^0^_CDK4_) using thermodynamic integration. The difference (ΔΔG^0^) of these two TI calculations, ΔG^0^_CDK2_ and ΔG^0^_CDK4_, quantifies the energetic contribution for selectivity that can be attributed to the positive fascaplysin charge (Figure 3). The His95-Nε-H conformer was chosen for the CDK4 TI simulations, so we do not account for any contribution of a possible His95-Nδ-H hydrogen bond to fascaplysin and its potential effect on selectivity in these simulations. Hence, the change in free energy we derive from our TI Δsimulations is a reflection of the differential stabilisation of the positive fascaplysin charge The TI simulations were run for 19 values of λ for 5 ns each. These runs combined results from 25 data points (representing 200 ps windows each) for both, ΔG^0^_CDK4_ and ΔG^0^_CDK2_, respectively (Figure 7). The free energy for the transformation of CRB into FAS in the CDK2 and CDK4 complexes is subject to fluctuations, but both the curves are clearly separated all the time. Total ΔG^0^_CDK4_, the free energy for the CRB to FAS transformation in the CDK4 complex is 23.2±0.4 kcal/mol compared to 24.6±0.4 kcal/mol for ΔG^0^_CDK2_ in the CDK2 complex (errors from badge averaging). The effect of the positive charge in fascaplysin (or more precisely, the isoelectronic substitution of a neutral carbon with a positively charged nitrogen) is different in CDK2 and CDK4. In relative terms the accommodation of the positive charge is less costly in CDK4 than in CDK2. The positive charge on fascaplysin contributes with a ΔΔG^0^ of 1.4±0.6 kcal/mol to preferential binding to CDK4, corresponding to a factor of ca. 10 in terms of K_D_. While this result is not fully explaining the extraordinary difference in binding properties, it is clear that the positive inhibitor charge contributes substantially to the selectivity of fascaplysin to CDK4. This has important implications for the design of fascaplysin derived CDK4 inhibitors, a positive charge should be kept in derivatives. Interestingly, the highly specific CDK4 inhibitor PD0332991 [27] bears a tertiary amine and hence also a positive charge. It may achieve, at least partially, its specificity also via differential stabilisation of the positive charge.

## Conclusions

The molecular modelling study in this work addresses the remarkable selectivity of fascaplysin for CDK4. We have established a ‘hybrid model’ approach for CDK4 as a suitable starting point for ligand docking and molecular dynamics studies. Thermodynamic integration focussing on the effect of the positive charge on fascaplysin demonstrates that this charge significantly contributes to fascaplysin selectivity, while additional factors such as the polar interaction with His95^CDK4^ may also play a role. Molecular dynamics simulations indicate that the molecular basis of this effect may be due to an unfavourable interaction with Lys89^CDK2^. Our study suggests that there is a significant gain in specificity to be made by incorporating/maintaining a positively charged functional group when designing inhibitors selective for CDK4.

## Supporting Information

File S1 **PDB-file for the CDK4 hybrid model.** The model was generated as described in the methods section.
(PDB)
